# Effects of controlled artificial incubation on hatching success, morphometric traits, and early physiological indicators in olive ridley sea turtles (*Lepidochelys olivacea*) from Banyuwangi coast, Indonesia

**DOI:** 10.14202/vetworld.2026.2105-2116

**Published:** 2026-05-18

**Authors:** Ragil Angga Prastiya, Suherni Susilowati, Imam Mustofa, Aditya Yudhana, Ryan Adi Taufiqurrahman, Muhammad Athalah Daffa, Samira Musa Sasi, Nagia Musa Alghoul, Wiyanto Haditanojo

**Affiliations:** 1 Doctoral Program of Veterinary Science, Faculty of Veterinary Medicine, Universitas Airlangga, Indonesia; 2 Veterinary Medicine Study Program, Department of Health and Life Sciences, Faculty of Health, Medicine and Life Sciences (FIKKIA), Universitas Airlangga, East Java, Indonesia; 3 Research Group of Animal Biomedical and Conservation, Universitas Airlangga, Indonesia; 4 Faculty of Veterinary Medicine, Universitas Airlangga, Surabaya, East Java, Indonesia; 5 Veterinary Medicine Study Program, Faculty of Health, Medicine and Life Sciences (FIKKIA), Universitas Airlangga, East Java, Indonesia; 6 Zoology Department, Faculty of Science, University of Tripoli, Tripoli, Libya; 7 Libyan Center of Biotechnology Research, Tripoli, Libya; 8 Banyuwangi Sea Turtle Foundation (BSTF), East Java, Indonesia

**Keywords:** artificial incubation, conservation management, hatchling morphometrics, hatching success, Indonesia, *Lepidochelys olivacea*, physiological development, yolk absorption

## Abstract

**Background and Aim:**

Olive ridley sea turtles, *Lepidochelys olivacea*, are globally threatened marine reptiles whose reproductive success is strongly influenced by environmental conditions at their nests. Artificial incubation has been increasingly implemented to reduce egg mortality from predation, flooding, and anthropogenic disturbances; however, information on its effects on hatchling morphology and early physiological development remains limited. This study aimed to evaluate the effects of controlled artificial incubation using the Intan Room system on hatching success, hatchling morphometric traits, and early physiological indicators of olive ridley sea turtles originating from different nesting beaches along the Banyuwangi coast, Indonesia.

**Materials and Methods:**

Eggs collected from three nesting beaches in Banyuwangi, East Java, Indonesia, were relocated and incubated under controlled conditions using the Intan Room system. Incubation temperature and relative humidity were continuously monitored and maintained at approximately 28.6°C and 80%, respectively. Hatching success and incubation duration were recorded for all clutches. Hatchling morphometric parameters, including head dimensions, carapace measurements, plastron dimensions, and flipper lengths, were measured using digital calipers. Yolk absorption dynamics were assessed at fixed intervals after hatching as an indicator of early physiological development. Data were analyzed using descriptive statistics, one-way analysis of variance, Tukey’s post hoc test, and Pearson correlation analysis.

**Results:**

Controlled incubation maintained relatively stable thermal conditions within the optimal embryonic developmental range. The overall mean hatching success reached 83.7%, with beach-specific values ranging from 77.6% to 91.4%. Hatchlings from different nesting origins differed significantly in several morphometric parameters, including carapace length, plastron dimensions, and forelimb length (p < 0.05). Hatchlings originating from Sobo Beach generally demonstrated larger morphometric measurements. Yolk sac diameter progressively decreased during the first 9 h post-hatching, and yolk absorption dynamics showed significant positive correlations with carapace length, carapace width, and foreflipper length (p < 0.01).

**Conclusion:**

Controlled artificial incubation using the Intan Room system effectively supported high hatching success under stable thermal conditions. Nevertheless, morphometric and early physiological variation persisted among hatchlings from different nesting origins, indicating that pre-incubation biological factors continue to influence developmental outcomes despite environmental standardization. These findings emphasize the importance of incorporating nest provenance and standardized egg handling protocols into hatchery management programs to improve sea turtle conservation strategies.

## INTRODUCTION

Sea turtles are long-lived marine reptiles that play important roles in coastal and pelagic ecosystems through nutrient cycling and habitat structuring [1, 2]. Indonesia hosts six of the seven extant sea turtle species, most of which are classified as threatened and included in the Convention on International Trade in Endangered Species of Wild Fauna and Flora (CITES) Appendix I, reflecting the high conservation concern associated with their declining populations [3]. Among these species, the olive ridley sea turtle (*Lepidochelys olivacea*) is widely distributed along tropical coastlines, including the southern coast of Java, but continues to experience population declines driven by egg harvesting, habitat degradation, and climate-related environmental change [4–6]. Reproductive success in sea turtles is strongly influenced by nest microenvironmental conditions, particularly temperature and moisture, which regulate embryonic development, incubation duration, and hatchling phenotype [7]. Deviations from optimal thermal conditions may increase embryonic mortality, alter hatchling morphology, and disrupt temperature-dependent sex determination, thereby affecting long-term population dynamics [8]. Artificial incubation has been increasingly implemented as a conservation intervention to reduce nest loss caused by predation, flooding, and human disturbance [9]. In Indonesia, hatchery-based programs have primarily emphasized hatching success as a key indicator of conservation effectiveness [10]. However, hatching success alone does not necessarily reflect hatchling quality or post-emergence fitness. Post-hatching traits such as body size, limb development, and yolk absorption efficiency are critical determinants of early survival, yet remain poorly documented in most hatchery-based studies of olive ridley sea turtles [11]. Moreover, few studies have evaluated whether hatchling morphological variation persists under standardized artificial incubation conditions, particularly when eggs originate from different nesting beaches. This limitation restricts understanding of the relative contributions of maternal origin and incubation environment to hatchling development.

Artificial incubation has become one of the most widely applied conservation strategies for protecting sea turtle eggs from natural and anthropogenic threats, including predation, tidal inundation, erosion, and human disturbance [12]. By regulating temperature and humidity, artificial incubation systems can substantially improve hatching success under unfavorable environmental conditions [13, 14]. Nevertheless, concerns remain regarding the ability of artificial incubation environments to accurately replicate the natural developmental conditions required for optimal embryogenesis and hatchling fitness. Previous studies have suggested that incubation conditions may influence hatchling morphology, locomotor performance, physiological condition, and energy utilization during the early post-hatching period [15]. Therefore, the evaluation of artificial incubation systems should extend beyond hatching success alone to include assessments of hatchling biological quality and developmental indicators.

Despite extensive research on incubation duration and hatching success, comparatively fewer studies have examined post-hatching traits, such as morphometric characteristics and physiological condition, which are closely associated with early survival capacity [16]. Most previous studies have been laboratory-based or conducted under semi-natural hatchery conditions, potentially overlooking maternal-origin effects and early microenvironmental influences that may persist even after egg relocation [17–19]. Furthermore, studies evaluating the effectiveness of automated incubation systems under tropical field conditions remain scarce, particularly in Indonesia. Consequently, the extent to which standardized artificial incubation conditions can minimize developmental variation among hatchlings from different nesting origins remains insufficiently understood.

Recent conservation initiatives in Indonesia have introduced automated artificial incubation systems, including the Intan Room developed by the Banyuwangi Sea Turtle Foundation (BSTF), which employs controlled temperature and humidity to stabilize embryonic development. Preliminary observations have reported relatively high hatching success using this system; however, comprehensive evaluations of hatchling biological quality, especially morphometric traits and early physiological indicators, remain limited [20]. In addition, climate change continues to intensify conservation challenges by altering nest thermal regimes and potentially disrupting temperature-dependent sex determination, thereby influencing long-term population structure and recruitment dynamics [21–23]. Although artificial incubation has been proposed as a potential mitigation strategy through environmental regulation, its effects on hatchling morphology and physiological readiness under tropical coastal conditions remain inadequately characterized [24].

Current knowledge regarding artificial incubation in sea turtles is largely focused on incubation duration and hatching success, whereas comparatively little information is available on hatchling quality, morphometric variation, and early physiological development following controlled incubation. Existing studies rarely integrate morphometric assessments with physiological indicators such as yolk absorption dynamics, despite their importance in determining hatchling vigor and post-emergence survival potential. In addition, the persistence of developmental variation among hatchlings originating from different nesting beaches under identical incubation conditions remains poorly understood. Most previous investigations have also relied on semi-natural hatchery systems or laboratory simulations, limiting the applicability of findings to field-based conservation programs operating under tropical environmental conditions. Consequently, there remains a significant gap in understanding whether controlled incubation systems can standardize hatchling developmental outcomes or whether pre-incubation biological factors continue to influence hatchling phenotype despite environmental regulation.

Therefore, this study aimed to evaluate the effects of controlled artificial incubation using the Intan Room system on incubation stability, hatching success, hatchling morphometric characteristics, and early physiological indicators of *L. olivacea*) originating from different nesting beaches along the Banyuwangi coast, Indonesia. Specifically, the study investigated thermal incubation profiles, incubation duration, hatchling morphometric variation, and yolk absorption dynamics under standardized incubation conditions. Furthermore, the study aimed to determine whether hatchling developmental differences persist among nesting origins despite environmental standardization within the artificial incubation system. The findings of this study are expected to provide region-specific scientific evidence to improve hatchery management practices and support evidence-based conservation strategies for olive ridley sea turtles in Indonesia and other tropical nesting regions.

## MATERIALS AND METHODS

### Ethical approval

All experimental procedures involving *L. olivacea* eggs and hatchlings were conducted in accordance with institutional and international ethical guidelines for wildlife research and conservation. Ethical approval for this study was obtained from the Ethics Committee of the Central Laboratory for Life Sciences, Universitas Brawijaya, Indonesia (Approval No. 113/LSIH/EP/2025), prior to the commencement of field and laboratory activities. All procedures complied with Indonesian wildlife conservation regulations and the provisions of the Convention on International Trade in Endangered Species of Wild Fauna and Flora (CITES) for protected marine species. Egg handling, transportation, incubation, and hatchling assessments were performed using non-invasive procedures designed to minimize physical disturbance, mechanical injury, and physiological stress. No invasive manipulations, surgical procedures, or harmful interventions were performed during the study. Hatchlings were handled only for essential morphometric and physiological assessments and were subsequently maintained under environmentally controlled conditions to ensure animal welfare standards throughout the experimental period.

### Study period and location

This study was conducted from May to December 2024 in Banyuwangi Regency, East Java, Indonesia, along the southern coastal region, which is characterized by important nesting habitats for olive ridley sea turtles (*Lepidochelys olivacea*). Artificial incubation activities were conducted at the BSTF in Sobo Village, Banyuwangi, Indonesia (8.2364° S, 114.3779° E). The incubation facility used the Intan Room system, a closed environmental chamber designed to maintain stable incubation temperature and relative humidity via automated thermo-hygrometric control.

An initial field survey was conducted across five nesting beaches representing different ecological and thermal microhabitats, namely Sobo Beach, Boom Beach, Pondok Nongko Beach, Pulau Santen Beach, and Cemara Beach. Sobo Beach was located adjacent to the BSTF conservation facility, whereas Boom Beach represented a semi-urban coastal environment. Pondok Nongko Beach was characterized as an isolated low-disturbance nesting site, Pulau Santen Beach exhibited near-lagoon sandy substrates, and Cemara Beach represented an open coastline with greater diurnal temperature variation. Following evaluation of clutch completeness and sampling suitability, only Boom Beach, Pondok Nongko Beach, and Pulau Santen Beach were included in the final analysis. These nesting beaches were located approximately 0.5–15 km from the artificial incubation facility.

Despite their close geographic proximity, these beaches exhibited variation in environmental characteristics, including sand composition, degree of anthropogenic activity, coastal hydrodynamics, and local microclimatic conditions, all of which may influence maternal nesting behavior and early egg conditions prior to relocation. To standardize post-oviposition incubation conditions, all collected eggs were transferred to a single controlled incubation facility. Egg relocation from natural nests to the artificial incubation chamber was conducted within 4–6 h after oviposition and never exceeded 12 h to minimize disruption of early embryonic development. Relocation procedures were performed before 10:00 AM or after 6:00 PM to avoid excessive thermal exposure. During transportation under high ambient temperatures, eggs were covered with damp cloths or leaves to maintain thermal stability and minimize overheating.

### Study design

This study employed an observational experimental design to evaluate the effects of controlled artificial incubation using the Intan Room system on hatching success, hatchling morphometric characteristics, and early physiological indicators of *L. olivacea* originating from different nesting beaches. Eggs collected from natural nesting sites were relocated into a controlled incubation environment where temperature and humidity were continuously regulated and monitored throughout the incubation period.

The experimental framework included assessment of incubation temperature stability, incubation duration, hatching success, hatchling morphometric parameters, and post-hatching yolk absorption dynamics. Morphometric comparisons were conducted among hatchlings originating from three nesting beaches under standardized incubation conditions to evaluate whether developmental variation persisted despite environmental regulation. In addition, yolk absorption dynamics were analyzed descriptively in a subset of hatchlings maintained under controlled post-emergence conditions to assess early physiological development and energy utilization.

### Controlled incubation using the Intan Room system

The Intan Room system consisted of a controlled incubation chamber equipped with automated thermo-hygrometric regulation systems designed to maintain stable embryonic developmental conditions. Incubation temperature was maintained between 27°C and 29°C, while relative humidity was regulated at approximately 80% to simulate optimal natural nesting conditions.

Environmental parameters were continuously monitored using HOBO® data loggers (Onset Computer Corporation, Bourne, MA, USA) programmed to record hourly throughout the incubation period. The incubation chamber was designed to minimize the influence of external environmental factors such as predators, rainfall, and rapid thermal fluctuations. Eggs were visually inspected twice daily for fungal contamination, shell desiccation, or developmental abnormalities.

### Egg collection and relocation

Freshly laid eggs were manually excavated from natural nests immediately after oviposition using sterile gloved hands to minimize contamination and mechanical damage. Eggs were transported in sand-filled containers to preserve egg orientation and structural stability during relocation. Egg rotation was strictly avoided throughout handling and transportation procedures to prevent disruption of early embryonic development.

Relocation procedures were completed within 4–12 h post-oviposition, during early-morning or late-afternoon periods, to reduce thermal stress. During transportation, eggs were covered with moist towels to buffer temperature fluctuations and maintain microclimatic stability.

No pre-relocation assessment of egg fertility, including candling or visual screening, was performed because prolonged handling could increase the risk of embryonic disturbance. Therefore, all collected eggs were incubated regardless of presumed fertility status, and developmental success was evaluated upon completion of incubation.

### Incubation setup and monitoring

Incubation environmental conditions were continuously recorded using HOBO® data loggers to maintain optimal developmental conditions throughout the study period. Temperature and relative humidity data were continuously monitored and subsequently summarized as daily averages to evaluate thermal and humidity stability.

Routine monitoring was conducted twice daily during morning and afternoon periods. Eggs were visually inspected during each observation to identify fungal contamination, shell collapse, surface desiccation, discoloration, or structural abnormalities indicative of impaired development.

Incubation duration under the Intan Room system was descriptively compared with incubation durations previously reported for natural nests under comparable environmental conditions. However, no parallel in situ natural nest controls were included in the present study.

Environmental control systems were manually adjusted when deviations from target conditions occurred. Corrective interventions, including ventilation adjustments and humidity regulation, were implemented when the incubation temperature exceeded 30°C or fell below 27°C.

### Yolk absorption assessment

Yolk absorption dynamics were evaluated as indicators of early post-hatching physiological development and energy utilization. Yolk sac diameter was measured immediately after hatchling emergence (0 h) using a digital caliper (Mitutoyo, Kawasaki, Japan) with 0.01 mm precision. Subsequent reductions in yolk sac diameter were monitored over time to evaluate yolk absorption progression.

A subset of hatchlings (n = 8) originating from a single clutch collected at Sobo Beach was selected to minimize inter-clutch variability and ensure uniform incubation background conditions. Hatchlings were maintained in a controlled holding environment at 28–29°C and approximately 80% relative humidity to simulate natural post-emergence conditions while minimizing environmental stress.

Yolk sac measurements were performed at fixed intervals of 0, 3, 6, and 9 h post-emergence. All measurements were conducted by the same observer to minimize inter-observer variation, and each measurement was repeated 3 times before calculating the mean value.

The total duration required for yolk sac absorption was documented for each hatchling. Due to a limited sampling design, yolk absorption analyses were conducted descriptively within the selected hatchling group and were not compared among nesting beaches.

### Hatchling morphometric assessment

Hatchling morphometric assessment was performed to evaluate the effects of controlled artificial incubation on structural development. Immediately after hatching, morphometric measurements were obtained for head dimensions, carapace measurements, plastron dimensions, and flipper lengths.

For head morphometry, hatchlings were positioned in a standardized neutral posture on a flat surface. Head length was measured from the tip of the snout to the occipital region, whereas head width was measured at the widest region of the skull. All measurements were obtained using a digital caliper (Mitutoyo, Kawasaki, Japan) with 0.01 mm precision.

Carapace morphometric parameters included Straight Carapace Length (SCL), curved carapace length, and carapace width. SCL was measured as the straight-line distance between the anterior edge of the nuchal scute and the posterior edge of the supracaudal scute using digital calipers. Curved carapace length was measured along the natural curvature of the carapace using a flexible measuring tape. Carapace width was measured across the widest lateral margins of the carapace.

Plastron length was measured along the midline from the anterior gular scutes to the posterior anal scutes. Plastron width was measured across the widest region of the plastron, typically across the humeral or pectoral scutes.

Flipper morphometric assessment included measurements of foreflipper and hindflipper lengths. Foreflipper length was measured from the shoulder joint to the distal tip of the flipper, whereas hindflipper length was measured from the hip joint to the distal tip of the flipper. Bilateral measurements were obtained, and visible morphological abnormalities were recorded descriptively.

Each morphometric parameter was measured three times per hatchling, and the mean was used for statistical analysis to reduce measurement error. All morphometric measurements were performed by the same observer to minimize inter-observer variability.

Hatchlings analyzed in this study originated from multiple clutches collected from the three nesting beaches included in the study. The number of hatchlings evaluated per group is reported in the Results section.

### Data collection and statistical analysis

Temperature data recorded hourly using HOBO® data loggers (Onset Computer Corporation, Bourne, MA, USA) were analyzed descriptively to evaluate thermal stability during incubation. Incubation duration and hatching success were summarized using descriptive statistics. Hatching success was calculated as the percentage of successfully hatched eggs relative to the total number of incubated eggs per clutch.

Morphometric variables were analyzed using one-way analysis of variance (ANOVA) to evaluate differences among nesting beach origins. Prior to statistical analysis, data normality and homogeneity of variances were assessed using the Shapiro–Wilk and Levene’s tests, respectively. When significant differences were identified (*p* < 0.05), Tukey’s post hoc test was applied for pairwise group comparisons.

Associations between yolk absorption dynamics and hatchling morphometric parameters were evaluated using Pearson correlation analysis. Statistical analyses were performed using IBM SPSS Statistics version 28.0 (IBM Corp., Armonk, NY, USA), and results are presented as mean ± SD.

## RESULTS

### Incubation temperature profile

**Figure 1:**
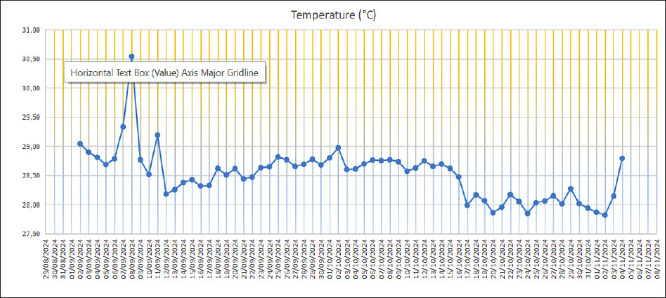
Temperature changes during the incubation period of olive ridley sea turtle (*Lepidochelys olivacea*) eggs for 64 days under the Intan Room incubation system.

Throughout the incubation period, the temperature in the Intan Room incubation system remained relatively stable, with no major fluctuations. Recorded temperatures ranged from 27.8°C to 30.5°C, with an overall mean temperature of 28.7 ± 0.4°C. Minor transient temperature increases above 30.0°C were occasionally observed for short durations (Figure 1). Relative humidity was consistently maintained at approximately 80% throughout the incubation period. No consistent thermal fluctuations were detected among clutches originating from different nesting beaches.

Near the end of the incubation period, the temperature returned to the optimal developmental range, indicating stabilization of the incubation environment within the controlled chamber.

### Hatching success and incubation duration

A total of nine clutches were relocated and incubated under controlled conditions using the Intan Room system, with three clutches representing each nesting beach. Each clutch contained approximately 80–110 eggs. Hatching success was calculated as the number of successfully hatched eggs relative to the total number of incubated eggs because no pre-incubation fertility assessment was performed.

The overall mean hatching success was 83.7%, whereas beach-specific hatching success ranged from 77.6% to 91.4%. Significant variation in hatching success was observed among nesting beach origins (one-way ANOVA, p < 0.01).

The incubation period was identical across all clutches, with hatchling emergence occurring 64 days after oviposition. No variation in incubation duration was observed among nesting beaches. Incubation duration was defined as the number of days from oviposition until first hatchling emergence. The uniform incubation duration reflected the stable thermal environment maintained within the Intan Room system.

### Hatchling morphometric characteristics

In total, 45 hatchlings were evaluated morphometrically under controlled incubation conditions. Hatchlings originated from three nesting beaches, namely Boom Beach (n = 15), Pondok Nongko Beach (n = 15), and Pulau Santen Beach (n = 15). Morphometric assessment included measurements of head, carapace, and plastron dimensions, as well as fore- and hindflipper measurements.

A unique color-coded rope marker was assigned to each hatchling to maintain consistent identification across repeated assessments and prevent duplicate measurements.

There were significant differences in several hatchling morphometric parameters among nesting beach origins (Table 1). Significant variation was observed in SCL, curved carapace length, carapace width, plastron dimensions, and several flipper measurements (p < 0.05). Hatchlings originating from Sobo Beach generally exhibited larger morphometric measurements than hatchlings from Boom Beach and Pondok Nongko Beach.

**Table 1:** Morphometric comparison of hatchling olive ridley sea turtles (*Lepidochelys olivacea*) originating from Pondok Nongko Beach, Boom Beach, and Sobo Beach.

Parameter	Pondok Nongko Beach (Mean ± SD)	Boom Beach (Mean ± SD)	Sobo Beach (Mean ± SD)	p-value
Head length	24.77 ± 2.02	22.49 ± 3.82	26.24 ± 1.58	< 0.001
Head width	13.99 ± 0.72	14.53 ± 1.54	14.36 ± 1.82	NS
Carapace length	34.87 ± 8.66	38.88 ± 4.33	39.82 ± 3.57	0.002
Carapace width	29.10 ± 4.86	32.33 ± 3.42	29.73 ± 4.95	0.001
Plastron length	27.43 ± 4.25	32.25 ± 6.19	28.26 ± 4.25	< 0.001
Plastron width	26.86 ± 2.10	30.12 ± 3.18	27.38 ± 2.16	< 0.001
Right foreflipper length	35.68 ± 7.11	34.92 ± 2.22	37.38 ± 3.33	< 0.001
Right foreflipper width	11.15 ± 1.78	11.70 ± 1.43	11.65 ± 0.96	NS
Right hindflipper length	35.50 ± 6.32	33.92 ± 3.41	37.87 ± 3.37	NS
Right hindflipper width	11.90 ± 4.31	12.12 ± 1.58	11.94 ± 4.17	NS
Left foreflipper length	20.39 ± 3.79	17.39 ± 2.98	23.82 ± 22.87	< 0.001
Left foreflipper width	11.15 ± 2.10	11.87 ± 1.75	10.96 ± 2.45	NS
Left hindflipper length	18.55 ± 2.62	16.96 ± 3.08	20.06 ± 2.61	< 0.001
Left hindflipper width	11.66 ± 2.31	12.03 ± 2.07	11.76 ± 2.28	NS

**Figure 2:**
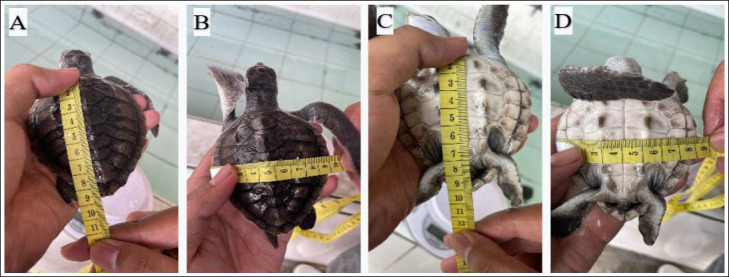
Morphometric assessment of olive ridley sea turtle (*Lepidochelys olivacea*) hatchlings. (A) Measurement of carapace length; (B) measurement of carapace width; (C) measurement of plastron length; and (D) measurement of plastron width.

Significant differences were also identified in head morphometric parameters among hatchlings from different nesting beaches (p < 0.05). Hatchlings from Sobo Beach exhibited greater mean head dimensions than those from Boom Beach and Pulau Santen Beach. Plastron measurements similarly demonstrated significant variation among nesting origins (p < 0.05). Foreflipper length differed significantly among beach groups (p < 0.01), whereas hindflipper length did not show statistically significant variation among nesting beaches (p > 0.05). Tukey’s Honestly Significant Difference post hoc analysis indicated that the greatest morphometric differences for carapace- and flipper-related measurements occurred between hatchlings originating from Pondok Nongko Beach and Pulau Santen Beach.

### Post-hatching yolk absorption

Post-hatching yolk absorption was evaluated as an indicator of early physiological development and metabolic readiness in hatchlings incubated under controlled conditions. Residual yolk reserves serve as an important endogenous energy source during the early post-emergence period prior to active feeding behavior.

Yolk absorption dynamics were evaluated in eight hatchlings originating from a single clutch collected from Sobo Beach and incubated using the Intan Room system. Yolk sac diameter was measured at fixed intervals from emergence (0 h) until 9 h post-hatching.

**Figure 3:**
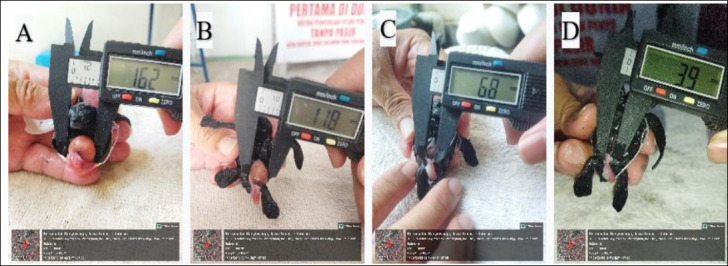
Visual comparison of yolk sac diameter in olive ridley sea turtle (*Lepidochelys olivacea*) hatchlings at different post-emergence intervals using a digital caliper. (A) Yolk diameter at 0 h; (B) yolk diameter at 3 h; (C) yolk diameter at 6 h; and (D) yolk diameter at 9 h.

A progressive reduction in yolk sac diameter was observed throughout the monitoring period (Figure 3). The greatest reduction occurred during the first 6 h after hatching, whereas the absorption rate slowed between 6 and 9 h post-emergence.

**Table 2:** Yolk diameter measurements (mm) at different post-hatching observation intervals.

Sample	Yolk diameter (0 h)	Yolk diameter (3 h)	Yolk diameter (6 h)	Yolk diameter (9 h)
Without rope	15.5	7.5	6.6	5.9
Yellow rope	16.9	13.5	8.2	6.9
Blue and white rope	11.8	9.3	6.8	5.3
Black rope	9.7	7.7	5.9	4.4
Brown rope	14.5	10.4	6.7	5.7
Blue rope	9.8	8.1	6.4	5.3
Yellow and white rope	8.3	8.1	6.1	4.8

Pearson correlation analysis demonstrated significant positive associations between yolk absorption dynamics and several hatchling morphometric parameters. Yolk absorption rate showed strong positive correlations with SCL (r = 0.86, p = 0.006), carapace width (r = 0.82, p = 0.009), and foreflipper length (r = 0.84, p = 0.007). In contrast, no significant correlations were identified between yolk absorption rate and head width (r = 0.41, p = 0.32) or hindflipper length (r = 0.38, p = 0.36).

## DISCUSSION

### Incubation temperature stability and hatchling development

The present study demonstrated that incubation temperature stability and nest origin collectively influenced hatchling morphology and early physiological indicators in *L. olivacea*. Temperature profiles within the Intan Room remained largely within the recognized optimal range for tropical sea turtle embryogenesis (28–30°C), which is associated with stable metabolic activity and normal organ development [25]. Brief deviations above 30.5°C or below 27.8°C were infrequent and short-lived, indicating operational sensitivity rather than chronic thermal stress. Although these fluctuations did not result in overt developmental failure, they may have contributed to subtle variation in yolk utilization efficiency and hatchling morphometrics, consistent with previous reports on thermal effects during embryogenesis [26].

### Influence of nest origin on morphometric outcomes

Nest origin significantly influenced morphometric outcomes despite uniform incubation conditions. This finding indicates that biological factors acting before relocation, such as maternal provisioning or early microenvironmental exposure, remained detectable after incubation standardization rather than implying direct genetic causation [27]. Higher mean values of head length, carapace length, and plastron width in hatchlings from Sobo Beach suggest differences in initial egg characteristics, such as yolk volume or hormonal content, that may influence developmental trajectories. These results support the persistence of maternal or site-related effects but do not allow direct inference regarding genetic differentiation because molecular data were not included [28].

### Limb morphometry and developmental heterogeneity

Differences in limb morphometrics, particularly in the left foreflipper, further indicate developmental heterogeneity among hatchlings from different nesting origins. Limb development in reptiles is known to be sensitive during mid-to-late embryogenesis, and even minor thermal instability can influence muscle and skeletal differentiation [29]. The asymmetries observed in this study were subtle and remained within normal morphological ranges, but they may reflect differential growth trajectories rather than pathological development. Although limb dimensions are often associated with locomotor performance, no direct behavioral or performance assessments were conducted; therefore, the functional implications should be interpreted with caution [30].

### Yolk absorption and early physiological condition

Variation in yolk absorption rate provides additional insight into early post-hatching physiological condition. Hatchlings exhibiting slower yolk absorption may have experienced delayed metabolic activation, whereas individuals completing absorption more rapidly may have been physiologically more prepared for early post-emergence demands [31]. The observed associations between yolk absorption dynamics and morphometric traits suggest coordinated energy utilization rather than a direct causal relationship, particularly because yolk measurements were limited to a subset of hatchlings from a single nesting beach [32]. These findings highlight yolk absorption as a potentially informative but underutilized indicator of early hatchling condition.

### Interpretation of artificial incubation outcomes

Collectively, the data indicate that artificial incubation alone does not homogenize hatchling phenotypes. Despite controlled temperature and humidity, morphometric and physiological variation persisted among hatchlings from different nesting beaches, emphasizing the role of pre-incubation biological factors. This pattern aligns with evidence that sea turtle embryonic development reflects the interaction between incubation environment and maternal investment rather than environmental control alone [33].

### Conservation and hatchery management implications

From a conservation management perspective, the results suggest that artificial incubation programs should not rely solely on thermal regulation to optimize hatchling quality. Incorporating nest provenance records and standardized egg handling protocols may improve interpretation of hatchery outcomes and reduce unintended bias in hatchling production [34]. Importantly, this study did not include direct comparisons with in situ natural nests; therefore, conclusions are limited to variation within artificial incubation conditions rather than comparative performance against natural incubation systems.

### Limitations and future scope

Several limitations should be acknowledged. The controlled incubation environment reduced natural microclimatic variability and may not fully represent the range of conditions experienced in natural nests [35]. The study was limited to three nesting beaches within a single region, restricting broader ecological inference [36]. Post-hatching assessments were short-term and did not include hatchling mass, locomotor performance, orientation behavior, or sex ratio determination, all of which are important indicators of fitness and conservation relevance [37]. Incubation duration showed no observable variation among clutches, which may reflect the relatively narrow thermal range maintained in the controlled system and should be interpreted within this experimental context. In addition, genetic differentiation among nesting beaches was not assessed, limiting the interpretation of heritable effects.

Future studies should incorporate broader spatial sampling, genetic or maternal trait analyses, hatchling mass assessment, locomotor performance testing, orientation behavior evaluation, sex ratio determination, and post-release monitoring to better link hatchling morphology and physiology with early survival and recruitment [38]. Longitudinal assessments would strengthen the understanding of how early-life traits influence population dynamics. Ethical considerations were upheld by adhering to wildlife-handling regulations and non-invasive measurement protocols. The findings also support community-based conservation initiatives by emphasizing careful egg handling, provenance documentation, and standardized hatchery practices [39, 40].

## CONCLUSION

Controlled artificial incubation using the Intan Room system successfully maintained relatively stable thermal and humidity conditions throughout the incubation period and achieved a comparatively high mean hatching success of 83.7% in *L. olivacea*. The incubation environment remained largely within the optimal embryonic developmental range, supporting consistent incubation duration across all clutches. Nevertheless, significant variation in hatchling morphometric characteristics persisted among nesting beach origins despite standardized incubation conditions. Hatchlings originating from Sobo Beach generally exhibited greater head dimensions, carapace measurements, and several flipper-related morphometric traits than hatchlings from other nesting beaches. In addition, yolk absorption dynamics demonstrated significant positive associations with SCL, carapace width, and foreflipper length, indicating a relationship between early physiological development and hatchling structural characteristics.

The findings suggest that artificial incubation systems can effectively enhance hatching output under controlled environmental conditions; however, environmental standardization alone may not eliminate developmental variation among hatchlings. Pre-incubation biological factors, including maternal provisioning, egg characteristics, and early nesting microenvironmental influences, likely continue to affect embryonic development and hatchling phenotype after egg relocation. These observations emphasize that hatchling quality assessment should extend beyond hatching success and include morphometric and physiological indicators that may influence early survival potential.

From a practical conservation perspective, the study provides region-specific evidence supporting the application of controlled artificial incubation systems in tropical sea turtle conservation programs. The results highlight the importance of maintaining stable incubation conditions while incorporating nest-provenance documentation and standardized egg-handling protocols into hatchery management practices. Such approaches may improve the interpretation of hatchery performance and reduce unintended developmental variation among hatchlings. The study also supports the value of community-based hatchery systems, such as those implemented by the BSTF, in mitigating egg loss from predation, flooding, and anthropogenic disturbances in vulnerable nesting areas.

A major strength of the present study was the integration of hatchling morphometric assessment with early physiological evaluation under field-based controlled incubation conditions. Unlike many previous studies that focused primarily on hatching success, the present work incorporated yolk absorption dynamics as an additional indicator of early developmental condition. Furthermore, the use of a standardized automated incubation system allowed evaluation of hatchling variation under relatively stable environmental conditions, thereby improving understanding of the influence of nesting origin on developmental outcomes.

In conclusion, controlled artificial incubation using the Intan Room system represents a promising conservation strategy to improve hatching success in olive ridley sea turtles (*Lepidochelys olivacea*) under tropical field conditions. However, developmental variation in hatchlings persisted despite environmental standardization, indicating that pre-incubation biological factors continue to influence hatchling morphology and early physiological condition. Therefore, successful hatchery management should integrate both environmental regulation and biological provenance considerations to optimize hatchling quality and strengthen long-term sea turtle conservation outcomes.

## DATA AVAILABILITY

The supplementary data can be made available from the corresponding author upon request.

## AUTHOR’S CONTRIBUTIONS

RAP: Conceptualized and designed the study, conducted field and laboratory data collection, performed data analysis and interpretation, and prepared the original manuscript draft. SS and IM: Participated in study design, supervised the histological analyses, and contributed to data interpretation. AY: Assisted with statistical analysis, data visualization, and manuscript revision. RAT and MAD: Conducted field sampling, incubation monitoring, and laboratory data collection. SMS and NMA: Contributed to data curation, statistical analysis, and interpretation of results. WHT: Provided the conceptual framework for the conservation strategy and critically revised the manuscript. All authors reviewed and approved the final manuscript.
